# A comparative analysis of COVID-19 seroprevalence rates, observed infection rates, and infection-related mortality

**DOI:** 10.3389/fpubh.2025.1504524

**Published:** 2025-01-28

**Authors:** Eric W. Ford, Kunal N. Patel, Holly Ann Baus, Shannon Valenti, Jennifer A. Croker, Robert P. Kimberly, Steven E. Reis, Matthew J. Memoli

**Affiliations:** ^1^School of Public Health, University of Alabama at Birmingham, Birmingham, AL, United States; ^2^College of Health and Human Sciences, Northern Illinois University, Dekalb, IL, United States; ^3^Clinical Studies Unit, Laboratory of Infectious Diseases, National Institute of Allergy and Infectious Diseases, National Institutes of Health, Bethesda, MD, United States; ^4^Clinical and Translational Science Institute, University of Pittsburgh, Pittsburgh, PA, United States; ^5^Center for Clinical and Translational Science, School of Medicine, University of Alabama at Birmingham, Birmingham, AL, United States

**Keywords:** COVID-19 surveillance, seroprevalence, COVID-19 mortality risk, COVID-19 disparities, COVID-19 regional differences

## Abstract

**Objectives:**

The COVID-19 pandemic highlighted the need for data-driven decision making in managing public health crises. This study aims to extend previous research by incorporating infection-related mortality (IRM) to evaluate the discrepancies between seroprevalence data and infection rates reported to the Centers for Disease Control and Prevention (CDC), and to assess the implications for public health policy.

**Study design:**

We conducted a comparative analysis of seroprevalence data collected as part of an NIH study and CDC-reported infection rates across ten U.S. regions, focusing on their correlation with IRM calculations.

**Methods:**

The analysis includes a revision of prior estimates of IRM using updated seroprevalence rates. Correlations were calculated and their statistical relevance assessed.

**Results:**

Findings indicate that COVID-19 is approximately 2.7 times more prevalent than what CDC infection data suggest. Utilizing the lower CDC-reported rates to calculate IRM leads to a significant overestimation by a factor of 2.7. When both seroprevalence and CDC infection data are combined, the overestimation of IRM increases to a factor of 3.79.

**Conclusion:**

The study highlights the importance of integrating multiple data dimensions to accurately understand and manage public health emergencies. The results suggest that public health agencies should enhance their capacity for collecting and analyzing seroprevalence data regularly, given its stronger correlation with IRM than other estimates. This approach will better inform policy decisions and direct effective interventions.

## 1 Introduction

Since its emergence in late 2019, COVID-19, caused by the SARS-CoV-2 virus, has dramatically altered public health dynamics (1). Efforts to manage and control the disease’s spread varied greatly from place-to-place due to a lack of epidemiological insights about true infection rates and the infection-related mortality (IRM) (2–4). One key epidemiological measure is the seroprevalence rate, which refers to the proportion of a population with specific antibodies against a virus, that indicates the extent of an infection’s spread (5). Observed infection rates denote the number of confirmed cases over time, shedding light on both the spread and severity of a virus. Infection-related mortality (IRM) represents the number of deaths from a specific condition within a population, demonstrating the pathogen’s lethality and guiding strategies to reduce associated fatalities (6). Although these metrics are disparate in some respects, they are interrelated, offering critical insights into disease dynamics when taken together (7). However, lacking accurate infection rate estimates, may lead to misestimation of IRM (6, 8). Moreover, as the ultimate aim of any screening program is to reduce IRM, it is essential to collect the most accurate estimates of infection rates and related deaths (9).

The purpose of this research note is to replicate and extend the prior analysis of COVID-19 seroprevalence and reported infection rates to include IRM comparisons (10). Previously, the research team analyzed seropositivity in adults who had not previously been diagnosed with COVID-19 using quota sampling drawn from 241,424 volunteers to yield sufficient subpopulations to assess various regions (11) and demographic groups (*n* = 11,382) (ClinicalTrials.gov XXX). The seroprevalence-screening data was then combined with observed infection-rates and IRM information gathered by the Centers for Disease Control and Prevention (CDC) to compare and contrast seropositivity versus symptomatic infection-rate estimates’ utility in predicting IRM. Statistical analyses were conducted to estimate the IRM using both seroprevalence and CDC infection rate data sources.

The findings and discussion have utility for policymakers, public health organizations, and researchers. Policymakers can use the comparisons to better assess the necessity and effectiveness of the interventions implemented. As public health practitioners prepare for future epidemics, the study gives an example of what can be gained from seroprevalence studies (12). For researchers, the results provide information that informs future modeling of disease spread (13).

## 2 Methods

The analyses herein rely on data representing diverse demographics and geographical regions, intending to capture the multifaceted nature of COVID-19’s spread and impact. Data for the analyses and comparisons were drawn from one primary sample collection and two public sources related to COVID-19 infections and IRM. The primary data collection focused on seropositive testing among asymptomatic individuals to provide a clearer understanding of the true infection spread beyond symptomatic cases. This focus is critical for calculating infection-related mortality (IRM) more accurately, as it ensures the inclusion of undiagnosed cases, which are often omitted in symptomatic-based estimates.

This study extends a prior analysis by Kalish et al. (10) and evaluates seroprevalence, observed infection rates, and infection-related mortality (IRM) to identify discrepancies and inform public health policies. Data was collected from April to August of 2020 as part of an National Institutes of Health (NIH) study (ClinicalTrials.gov NCT04334954). The primary dataset includes 11,382 participants sampled from a pool of 462,949 volunteers. Data were collected via venipuncture and dried blood spot microsampling to measure SARS-CoV-2 antibodies using ELISA. Detailed sampling methodologies ensured representative demographics aligned with U.S. Census estimates through iterative quota sampling. The analysis was stratified by U.S. regions as defined by the Department of Health and Human Services (HHS). A detailed map of these regional groupings can be found at https://www.cdc.gov/cove/data-visualization-types/hhs-region-map.html.

Information on COVID-19 infection rates was downloaded from the CDC’s website^1^ on September 28, 2021 (14). On the same day, COVID-19 IRM was drawn from another of the CDC’s publicly available datasets^2^ (15). The aggregated COVID-19 infection and IRMs through August of 2020 drawn from the CDC datasets and merged with the seroprevalence data. Variables related to sex, age, race, ethnicity, and geographic region were included for all three measures of COVID-19 prevalence.

Correlations among the various measures of COVID-19 were calculated along with confidence intervals and significance tests. The IRM ratio using seroprevalence and CDC infection report data as the denominator were calculated and compared. In addition, a region-by-region calculation of the correlations was made to assess the seroprevalence versus CDC infection rate statistics accuracy. Analyses of the combined dataset were conducted in SPSS.

## 3 Results

The demographic breakdown for the seroprevalence study is as shown in Table 1. These data were used to calculate the seropositive percentages and risk ratios across different demographic and geographical variables replicating the earlier research. The two analyses differed slightly in some of the point estimates, but all significance levels were similar. The small differences in point estimates are likely attributable to the CDC having revised the tables in the period between the two teams downloading the data.

**Table 1:** Sampling frame and demographics.

	Participants, *n* (%)
Sex	
Male	4,318 (47.83)
Female	4,710 (52.17)
Age	
18–35	1,807 (20.02)
36–45	2,193 (24.29)
46–55	1,650 (18.28)
56–65	1,505 (16.67)
>65	1,873 (20.75)
Ethnicity	
Hispanic, Latino/a, or Spanish origin	1,495 (16.56)
Not Hispanic, Latino/a, or Spanish origin	7,532 (83.43)
Missing or did not answer	1 (0.01)
Race^a^	
White	7,280 (77.48)
Black or African American	921 (9.8)
American Indian or Alaska Native	220 (2.34)
Asian^b^	655 (6.97)
Pacific Islander^c^	29 (0.31)
Other	291 (3.1)
Region^d^	
1-CT, ME, MA, NH, RI, and VT	453 (5.02)
2-NY and NJ	721 (7.99)
3-DE, DC, MD, PA, VA, and WV	1,321 (14.63)
4-AL, FL, GA, KY, MS, NC, SC, and TN	1,591 (17.62)
5-IL, IN, MI, MN, OH, and WI	1,352 (14.98)
6-AR, LA, NM, OK, and TX	1,056 (11.7)
7-IA, KS, MO, and NE	385 (4.26)
8-CO, MT, ND, SD, UT, and WY	358 (3.97)
9-AZ, CA, HI, and NV	1,348 (14.93)
10-AK, ID, OR, and WA	443 (4.91)
Work site^e^	
Home	4,249 (43.01)
Work	2,846 (28.81)
Other	185 (1.87)
Not currently working	2,520 (25.51)
Missing or did not answer	79 (0.8)

^a Accounts for multiple races per individual. b Asian Indian, Chinese, Filipino, Japanese, Korean, Vietnamese, or other Asian. c Native Hawaiian, Guamanian or Chamorro, Samoan, or other Pacific Islander. d Territories are not accounted for: American Samoa, Commonwealth of the Northern Mariana Islands, Federated States of Micronesia, Guam, Marshall Islands, and Republic of Palau. e Accounts for multiple work sites per individual.^

The statistical analysis showed significant differences in seropositive percentages across different gender, age, ethnic, racial, and geographical categories. For instance, compared to white/non-Hispanic individuals, African American study participants were more likely to have COVID-19 antibodies. These results suggest that the risk of infection does not necessarily differ significantly across age groups or gender but is influenced also by ethnicity, race, and geographical location in the U.S.

The data showed that the reported infection rates significantly underestimate the actual infection rates across all regions – similar to later research findings (16). Appendix A highlights that the nationwide seroprevalence estimate was 3.88 percent, while the CDC’s reported infection rate was only 1.44 percent of the population, suggesting that the reported rates may underestimate the number of infections by a factor of 2.7. Furthermore, we observed considerable regional variations in the discrepancy between seroprevalence rates and reported infection rates, possibly reflecting differences in testing capacities across regions. These findings underscore the importance of integrating diverse datasets when tracking disease spread to ensure more accurate monitoring.

In each region (rows), the table highlights the differences between seroprevalence and CDC reported infection rates. The table illustrates that the two measures for tracking infections differ significantly. In particular, the CDC reported infection rate was significantly lower than the seroprevalence rate in every region. Comparing the nationwide totals, the CDC’s reported infection rate is 1.44 percent of the population compared to the Seroprevalence estimate of 3.88 percent. One interpretation of this finding is that the CDC reported infection rates underestimate the number of infections by 2.7 times. In addition, the difference in rates varied greatly from one region to the next (please see Tables 2, 3). These differences may be a result of testing capacity in the regions. Therefore, the question becomes which measure is a better proxy for IRM?

**Table 2:** Comparing deaths attributed to COVID-19, seropositivity, CDC reported infections, and IRM.

Regions	Population	Cumulative deaths reported to CDC/population, (% Pop.)	Seropositive cases/total, (% Pop.)^c,d^	Cumulative infections reported to CDC/population, (% Pop.)^c,d^	*z*-stat^g^	CI for IRM
1-CT, ME, MA, NH, RI, and VT	15,116,205	14,679 (0.1)	17/453 (3.75)	190,425 (1.26)	4.757***	0.033–0.035
2-NY and NJ	29,490,243	48,188 (0.16)	78/721 (10.82)	620,665 (2.1)	16.299***	0.014–0.015
3-DE, DC, MD, PA, VA, and WV	31,284,526	14,319 (0.05)	40/1321 (3.03)	327,196 (1.05)	7.081***	0.016–0.017
4-AL, FL, GA, KY, MS, NC, SC, and TN	67,210,142	23,388 (0.03)	50/1591 (3.14)	1,191,227 (1.77)	4.142***	0.011–0.012
5-IL, IN, MI, MN, OH, and WI	53,075,027	23,415 (0.04)	54/1352 (3.99)	616,119 (1.16)	9.725***	0.009–0.011
6-AR, LA, NM, OK, and TX	42,891,661	15,721 (0.04)	32/1056 (3.03)	642,980 (1.5)	4.095***	0.012–0.014
7-IA, KS, MO, and NE	14,244,666	2,952 (0.02)	10/385 (2.6)	154,340 (1.08)	2.869**	0.007–0.008
8-CO, MT, ND, SD, UT, and WY	12,372,167	2,464 (0.02)	19/358 (5.31)	109,663 (0.89)	8.924***	0.011–0.012
9-AZ, CA, HI, and NV	51,249,610	15,290 (0.03)	38/1348 (2.82)	803,761 (1.57)	3.656***	0.012–0.013
10-AK, ID, OR, and WA	14,515,034	2,108 (0.01)	12/443 (2.71)	98,952 (0.68)	5.185***	0.008–0.009
Total	331,449,281	14,679 (0.1)	350/9028 (3.876%)^e^	4,755,328 (1.435%)^e^	19.512***	
Infection-related mortality^a,b,f^			0.0126%	0.0341%		

^a^ IRM calculated using seroprevalence as base denominator: 0.049%/3.876% = 0.0126%. ^b^ IRM calculated using CDC reported infections rates as base denominator: 0.049%/1.435% = 0.0341%. ^c^ Ratio of seropositive measurement to CDC cumulative infection count (3.876%/1.435%) = 2.7. ^d^ Ratio of CDC cumulative infection IRM to seropositive IRM estimate (0.0341% / 0.0126%) = 2.7. ^e^ A combined estimate of COVID-19 infections would sum the seropositive and reported rates (3.876% + 1.435% = 5.311%). ^f^ The IRM for the combined COVID-19 related rates would be (0.049%/5.311% = 0.009%). ^g^ Significance levels: *p < 0.05; **p < 0.01; ***p < 0.001.

**Table 3:** Stratified analysis by region and ethnic groups with statistical significance.

Region	Ethnic group	Seropositive cases (%)	Reported infections (%)	IRM (%) (Significance)	Confidence interval for IRM
1. CT, ME, MA, NH, RI, VT	White	5.0	0.8	0.0165*	0.015–0.018
	African American	6.0	0.5	0.0125*	0.010–0.015
2. NY, NJ	White	6.7	2.0	0.0321**	0.031–0.033
	African American	10.0	2.0	0.0248**	0.023–0.026
3. DE, DC, MD, PA, VA, WV	White	6.0	1.0	0.0217*	0.020–0.023
	African American	12.5	1.0	0.0120*	0.011–0.014
4. AL, FL, GA, KY, MS, NC, SC, TN	White	3.75	1.0	0.0260*	0.024–0.028
	African American	5.0	1.0	0.0198*	0.017–0.022
	Hispanic	6.7	1.3	0.0214*	0.020–0.024
5. IL, IN, MI, MN, OH, WI	White	4.2	1.0	0.0203*	0.018–0.023
	African American	6.0	0.8	0.0145*	0.013–0.016
	Hispanic	8.0	1.5	0.0270**	0.025–0.029
6. AR, LA, NM, OK, TX	White	3.6	1.0	0.0158*	0.014–0.018
	African American	5.0	0.83	0.0183*	0.016–0.020
	Hispanic	5.0	1.0	0.0210*	0.019–0.023
7. IA, KS, MO, NE	White	3.0	0.83	0.0130*	0.012–0.014
	African American	5.0	0.75	0.0160*	0.015–0.018
	Hispanic	5.0	0.83	0.0183*	0.016–0.021
8. CO, MT, ND, SD, UT, WY	White	3.33	0.8	0.0108*	0.009–0.013
	African American	5.0	1.25	0.0143*	0.013–0.016
	Hispanic	8.33	1.33	0.0200*	0.018–0.022
9. AZ, CA, HI, NV	White	2.86	0.8	0.0114*	0.010–0.013
	African American	4.0	1.0	0.0175*	0.016–0.019
	Hispanic	8.0	1.2	0.0250**	0.023–0.028
10. AK, ID, OR, WA	White	3.75	0.625	0.0125*	0.011–0.014
	African American	7.5	0.833	0.0167*	0.015–0.019
	Hispanic	10.0	1.0	0.0220**	0.020–0.025

Significance levels: *p < 0.05; **p < 0.01; ***p < 0.001.

Table 2 presents an extended comparison of seropositivity, reported infection rates, and COVID-19 attributed deaths, indicating discrepancies and potential underestimations in reported infection rates due to not capturing asymptomatic infections. The data reveal a significant underrepresentation of actual infection rates by reported figures, suggesting that the true extent of the pandemic was considerably higher than what was being reported at the time. Notably, the seropositivity rates, indicating the percentage of individuals with COVID-19 antibodies, consistently exceed the infection rates reported to the CDC, underlining the existence of a substantial number of undiagnosed or asymptomatic cases. The difference factor is a conservative estimate because the seroprevalence study’s initial round of sampling *did not* include participants that reported having COVID-19 symptoms. Given the two sampling frames were mutually exclusive in their inclusion criteria (e.g., asymptomatic versus symptomatic subjects), combining the two rates is a reasonable and would put the number people having been infected in the U.S. at 5.31 percent in July of 2020.

Underestimating the true infection rate gives rise to the concomitant issue that IRM will be *overestimated* by a commensurate amount–in this case approximately 2.7 times more than the actual mortality rate comparing seroprevalence and CDC rates. If the larger estimate of combined COVID-19 asymptomatic and symptomatic screening for infections is used, the IRM drops to 0.009% (0.049% / 5.311% = 0.009%). Using the larger infection-rate as the denominator, the overestimation of the IRM rate would rise to a factor of 3.79 compared to the calculation made when using just the CDC’s reported infection rate.

To assess the impact of regional and ethnic variation on our findings, we conducted a stratified analysis of seroprevalence, reported infection rates, and IRM by region and ethnic groups (Table 3). The results reveal consistent patterns across most regions and demographic groups, with seroprevalence rates generally higher among African American and Hispanic populations compared to White populations. For example, in Region 4 (AL, FL, GA, KY, MS, NC, SC, TN), African Americans exhibited a seroprevalence rate of 5.0%, compared to 3.75% among White participants. Similarly, Hispanic populations in Region 9 (AZ, CA, HI, NV) showed a seroprevalence rate of 8.0%, compared to 2.86% for White populations.

The IRM values also varied by ethnicity, with African American and Hispanic populations consistently showing lower IRM compared to White populations when calculated based on seroprevalence data. These differences likely reflect a combination of factors, including demographic variations in testing access, exposure risk, and underlying health conditions. The stratified analysis confirms that the overall trends identified in the study—such as the correlation between seroprevalence and IRM—remain robust across diverse subpopulations.

Table 4 presents the correlations between different measures of infection rates (CDC reported rates and seroprevalence) and the IRM, presenting a nuanced understanding of the pandemic’s dynamics. The Pearson correlation coefficients reveal a stronger correlation between seroprevalence rates and IRM (0.838, *p* = 0.002) compared to the correlation between CDC reported infection rates and IRM (0.656, *p* = 0.039). This underscores the superior predictive power of seroprevalence data in estimating the true impact of COVID-19 on mortality, suggesting that seroprevalence rates, which account for both symptomatic and asymptomatic infections, provide a more comprehensive assessment of the pandemic’s lethality. Furthermore, the equally weighted composite rate shows an even higher correlation with IRM (0.861, *p* = 0.001), indicating the benefit of integrating multiple data sources for a more accurate estimation of COVID-19’s fatality rate. These findings highlight the limitations of relying solely on reported infection rates and emphasize the importance of serological studies in understanding and responding to the pandemic.

**Table 4:** Correlations between infection rate measures (CDC reported infection rates and seroprevalence rates) and deaths attributed to COVID-19.

	Proportion of regional deaths	
Variables	Pearson correlation coefficient	*p*-value
Infection rate reported to CDC	0.656	0.039
Seroprevalence rate	0.838	0.002
Equally weighted composite rate	0.861	0.001

## 4 Discussion

This paper’s analysis of COVID-19 seroprevalence, observed infection rates, and infection-related mortality (IRM) reveals critical insights into the pandemic’s dynamics and the effectiveness of population health strategies. The magnitude of difference in the seroprevalence and CDC infection rate estimates (approximately 2.7 to 3.79 times) is important. In the epidemic’s early stages, it was assumed that the virus was far more infectious and virulent and that few people who were infected would remain asymptomatic (17). With the benefit of hindsight, it appears that the early epidemiological models may have underestimated infection rates and overestimated the IRM of COVID-19 (18). *Post hoc* studies of variability in excess deaths (19) and life expectancy changes (20) attributable to COVID-19 further support the mortality overestimation concern. The significant and large correlation between seroprevalence rates and IRM, as opposed to the weaker association with reported infection rates, underscores the pivotal role of seroprevalence studies in understanding the pandemic’s true extent and threat to life. These findings are important for four reasons, each of which has significant implications for public health policy, epidemic modeling, and future research directions.

Firstly, the discrepancy between seroprevalence rates and reported infection rates highlights the challenge of relying solely on symptomatic-based clinical diagnoses to gauge the pandemic’s reach. The underestimation of infection rates and asymptomatic spread can lead to suboptimal resource allocation and preparedness in managing healthcare systems’ responses. The variation in testing capacities across regions, as reflected in the disparity between these rates, underscores the systematic necessity for a standardized, widespread testing strategy to ensure accurate surveillance and response mechanisms. Our findings advocate for integrating seroprevalence data into population health monitoring strategies to achieve a more accurate representation of the infection landscape, which is crucial for tailoring interventions and allocating resources effectively.

Secondly, the higher likelihood of seropositivity among African-Americans and the observed regional variations in seroprevalence and reported infection rates call for targeted population health interventions. These findings suggest that the risk of COVID-19 is not evenly distributed across populations, with certain demographic and geographic groups (21) facing higher exposure and transmission rates. These disparities likely reflect differences in testing access, public health infrastructure, and underlying social determinants of health. For instance, regions with higher seropositivity among African American populations may indicate disparities in exposure risk due to employment patterns, housing density, or access to healthcare services. Understanding these factors is critical for designing equitable public health strategies and improving resource allocation. Public health initiatives must therefore adopt an intersectional approach, recognizing and addressing the social determinants of health that contribute to these disparities. This includes focusing on community-level interventions and ensuring equitable access to testing, provision of healthcare services, and vaccination programs.

Thirdly, the findings underscore critical policy implications of overestimating IRM. Exaggerated IRM estimates can lead to misallocated healthcare resources, where regions with lower infection rates may stockpile resources unnecessarily while regions with higher actual infection rates face shortages. Moreover, inflated IRM values may distort public perception, potentially leading to undue panic or skepticism about public health messaging if later corrections are made. Epidemic models that rely on inaccurate mortality estimates risk producing flawed projections, influencing public health interventions such as lockdowns, resource distribution, and vaccination strategies. These issues highlight the need for incorporating seroprevalence data into epidemic models to ensure accurate and equitable decision-making. Furthermore, overestimations of IRM can disproportionately affect regions with lower testing capacities, exacerbating inequities in resource distribution and public health response.

Fourth, the robust correlation between seroprevalence rates and IRM also opens avenues for further research into the dynamics of COVID-19 transmission and immunity within populations. Investigating the factors that contribute to the observed discrepancies between seroprevalence and reported infection rates, such as asymptomatic transmission, testing access, and reporting practices, will be crucial. Additionally, longitudinal studies to monitor changes in seroprevalence over time can provide insights into the duration of immunity, the impact of vaccination campaigns, and the emergence of new variants.

Finally, there are limitations to relying on serological surveying with regards to the specificity and sensitivity of that self-administered test. Researchers must be mindful of these limitations. Fortunately, in this case, the serological survey results tracked regional deaths better than infection rates reported to the CDC (see Table 4), which may not always be the case in other contexts. Further, the correlation using a composite rate calculated with equal weighting of our serological data and reported cases of COVID-19 showed a notable improvement in its association with deaths attributed to the disease. Thus, integrating multiple data sources —such as those that have lesser reliability and/or validity than the gold standard—provides additional utility to researchers when modeling diseases. Thus, integrating multiple data sources—such as those that have lesser reliability and/or validity than the gold standard—provides additional utility to researchers when modeling diseases.

## 5 Conclusion

This study underscores the importance of leveraging seroprevalence data alongside reported, symptomatic-infection rates and mortality data to guide public health decision-making. The findings highlight the need for comprehensive, multi-faceted surveillance systems that can accurately capture the pandemic’s scope and inform targeted, equitable interventions (13, 22). Given the seroprevalence study *did not* include people under the age of 18, it is likely that the overestimation of IRM may have been even larger. Further research is warranted to explore the underlying causes of the discrepancies identified and to refine the methodologies for pandemic monitoring and response.

The findings also suggest a need for targeted intervention strategies catering to more vulnerable demographics, such as African Americans, who showed a higher likelihood of being seropositive. Public health initiatives should focus on community-level interventions to reduce disease transmission in higher-density living conditions, which may be common in certain racial and ethnic communities. Furthermore, the results illuminate the importance of an intersectional approach in public health policy-making, recognizing the disparities across different demographics and geographical regions in disease impact. Such an approach can inform the allocation of resources, implementation of social interventions, and structuring of health systems responses to manage the ongoing pandemic and future health crises.

One limitation of this study is the potential variability in seroprevalence values due to differences in test accuracy, including sensitivity and specificity. While the serological tests used in this study were validated to meet high standards of reliability, even small deviations in test performance can significantly impact prevalence estimates, particularly in populations with low infection rates. False positives may inflate seroprevalence estimates, whereas false negatives could underestimate true infection rates. This variability can also lead to discrepancies when comparing our findings with those from other studies that use different serological assays with varying performance characteristics. Additionally, self-administered testing may introduce user error, further affecting accuracy. To mitigate these issues, we cross-referenced seroprevalence rates with infection-related mortality (IRM) and CDC-reported infection rates, which provided a consistent pattern of correlations across regions and demographic groups. Nevertheless, future research should strive to standardize serological methods and incorporate adjustments for test accuracy to enhance the comparability and validity of seroprevalence studies. This standardization is particularly important for informing public health policies and understanding the true burden of infectious diseases.

## Data Availability

The raw data supporting the conclusions of this article will be made available by the authors, without undue reservation.

## Footnotes

^1^ https://covid.cdc.gov/covid-data-tracker/#datatracker-home

^2^ https://data.cdc.gov/Case-Surveillance/Weekly-United-States-COVID-19-Cases-and-Deaths-by-/pwn4-m3yp/about_data
